# Validation of IgA nephropathy diagnosis in the Swedish Renal Registry

**DOI:** 10.1186/s12882-024-03512-2

**Published:** 2024-03-04

**Authors:** Johanna Rehnberg, Mårten Segelmark, Jonas F. Ludvigsson, Louise Emilsson

**Affiliations:** 1 Department of Nephrology and Centre for Clinical Research, County Council of Värmland, Central Hospital Karlstad, Karlstad, Sweden; 2 School of Medical Science, University of Örebro, Örebro, Sweden; 3 Department of Endocrinology, Nephrology and Rheumatology, Skåne University Hospital, Lund, Sweden; 4 Department of Clinical Sciences, Lund University, Lund, Sweden; 5 Department of Medical Epidemiology and Biostatistics, Karolinska Institute, Stockholm, Sweden; 6 Department of Pediatrics, Örebro University Hospital, Örebro, Sweden; 7 Division of Digestive and Liver Disease, Department of Medicine, Columbia University Medical Center, New York, NY USA; 8 Division of Epidemiology and Public Health, School of Medicine, University of Nottingham, Nottingham, UK; 9 Nysäter Health Care Center, Centre for Clinical Research, County Council of Värmland, Nysäter, Sweden; 10 Department of General Practice and General Practice Research Unit (AFE), Institute of Health and Society, University of Oslo, Oslo, Norway

**Keywords:** Validation, Swedish Renal Registry, Kidney biopsy, IgA nephropathy

## Abstract

**Aim:**

The Swedish Renal Registry (SRR) is a unique national quality registry that monitors the clinical trajectory of patients with chronic kidney disease (CKD). We have validated the biopsy data registered in the SRR for IgA Nephropathy (IgAN) diagnosis.

**Methods:**

In total 25% of all patients (*n* = 142), registered with IgAN in the SRR after having performed a kidney biopsy during 2015–2019, were randomly selected. We obtained original biopsy and medical records for 139 (98%) patients. We evaluated the IgAN diagnosis using a standardized template, calculated its positive predictive value (PPV) with 95% confidence interval (CI) and reported clinical features at the time of diagnosis.

**Results:**

A histological and clinical diagnosis of IgAN was confirmed in 132 of the 139 patients, yielding a PPV of 95% (95% CI 90–98%). Median age was 46 years (range: 18–85) and the male:female ratio was 2.1:1. The median creatinine level was 123 µmol/L, with a corresponding estimated glomerular filtration rate (eGFR) level of 51 mL/min/1.73m^2^. Histological features of IgA deposits were seen in all patients, hypercellularity in 102/132 (77.2%), C3 deposits in 98/132 (72.4%) and C1q deposits in 27/132 (20.5%) of the cases.

**Conclusion:**

Validating data is not research per se, but continuous validation of medical registries is an important feature necessary to ensure reliable data and the foundation of good epidemiological data for future research. Our validation showed a high PPV (95%) for IgAN diagnosis registered in the SRR. Clinical characteristics were consistent with previous reports. The biopsy data in the SRR will be a valuable resource in future IgAN research.

## Introduction

### IgA nephropathy

IgA Nephropathy (IgAN) is the most common primary glomerulonephritis in the world, affecting both sexes and all ages. The clinical phenotype varies from asymptomatic and only detectable on urinary dipstick testing, to terminal kidney failure with hypertension and heavy proteinuria with consequent need of dialysis or kidney transplantation in 15–50% of the verified cases. Another classic symptom is episodes of macroscopic hematuria occurring when patients have upper airway or gastrointestinal infections. In some cases, most often in children, the kidney manifestations are accompanied with purpura and/or joint pain and abdominal pain. The condition is then referred to as IgA vasculitis (IgAV) or formerly Henoch-Schönlein syndrome [1–4].

Since there are no pathognomonic serum or urine markers, diagnosing IgAN requires a kidney biopsy. A central finding in the immunohistological picture is the depositions of IgA molecules. More specifically the involved IgA antibodies are of the subclass IgA1 and with a distinct defect in their galactosylation of the amino acids in the hinge region of the antibody. The aberrant galactosylation reveals new glycan parts to IgG antibodies, allowing them to form Gd (galactose deficient)-IgA-IgG immune complexes. The immune complexes reach the kidney microvascular glomeruli and the mesangial supportive tissue and spark local immune and complement systems activation. The inflammatory response leads to mesangial matrix increase, hypercellularity, endocapillary proliferation and depositions of C3, IgG and/or IgM.

The identification of IgA deposits with immunofluorescence is the main criterion for IgAN when assessing renal specimen [2, 4–6]. Other kidney conditions may also have IgA deposits, especially systemic lupus erythematosus (SLE)-associated nephritis, but this can usually be distinguished by the typical so-called “full-house” positive immunofluorescence staining for IgG, IgA, IgM, C1q, and C3 in SLE [7].

There are also non-kidney conditions, e.g., liver disorders and viral infections such as HIV and hepatitis, where IgA deposits may occur in the kidney. Such IgA deposits are sometimes referred to as Secondary IgAN but the concept is debated [8]. Post-infectious glomerulonephritis can also show IgA deposits, but usually with a different deposition pattern and a higher ratio of C3 than IgA [8].

Nowadays, the assessment of kidney biopsy material is often classified using the Oxford classification and the MEST score. The goal of the MEST scoring system is to identify specific pathological features that more accurately predict risk of progression of IgAN, enabling clinicians to improve individual patient prognostication. It is based on four histopathological features of IgAN: mesangial hypercellularity(M), endocapillary hypercellularity (E), segmental glomerulosclerosis (S) and tubular/interstitial atrophy (T). Furthermore, a fifth finding, crescents (C), has been included in the assessment. The scoring system has been validated in several studies including the European VALIGA cohort [9]. The E score does not seem to add as much prognostic information as a positive T score with irreversible damage associated with a high risk of progression, or a positive C score as a sign of a rapidly progressive course [10, 11].

In Sweden, the assessment of kidney biopsies is centralized to three pathology departments in Stockholm (Karolinska Institute Solna/Huddinge, Gothenburg (Sahlgrenska Hospital) and Malmö (Skåne University Hospital).

### Swedish Renal Registry

The Swedish Renal Registry (SRR) is a national quality register monitoring adults (≥ 18 years) with chronic kidney disease (CKD) in Sweden. It was launched in 2007 when three existing registers (Swedish Registry of Renal Replacement Therapy, SRAU, started already in 1991; the Swedish Dialysis Database, SDDB and The Chronic Renal Disease Registry) were merged.

All Swedish nephrology departments report to the SRR. The collected data include information on diagnosis, kidney function levels at different follow up periods, choice of renal replacement therapy, laboratory values, blood pressure and medication. This allows a detailed follow-up throughout the patient trajectory [12].

In 2015, the SRR also started to collect kidney biopsy data to monitor early diagnosis of CKD and capture clinical indication, complications, and pathological findings of performed biopsies.

This study aimed to validate this biopsy data and more specifically the IgAN diagnosis. As this is an observational process, we only handled the amount of medical chart extracts needed to assess if the registered information was correct. In the validation process we do not aim to compare the choice of different treatments or to predict outcome, but solely evaluate the quality of data in the SSR. Validation of register data is not research per se, but it enables us to ensure that reliable data is used in future research.

## Methods

### Subjects

The biopsy data in the SRR contains manually registered detailed medical data (web-based) from performed biopsies including the pathology diagnosis (classified according to SNOMED; Systemized Nomenclature of Medicine [13]). Some 563 patients had an IgAN or IgAV diagnosis after a kidney biopsy performed 2015–2019.

We requested a random sample of 25% (142 of total 563) of these patients. The patients were selected by enrolling every fourth registered case chronologically. After being provided with the personal identity number (PIN) of selected patients, as well as extracts from the SRR and information on the primary caregiver, we requested original biopsy records and patient charts from relevant departments. Using this information, we were able to review the histological descriptions in the biopsy reports and examine if clinical symptoms and laboratory measurements were consistent with an IgAN diagnosis, and with the registered data in the SRR.

### Validation of IgAN

To validate if the patients´ histological diagnosis and clinical presentation were consistent with an IgAN diagnosis, we used similar criteria as Jarrick et al. in their previous validation of IgAN diagnosis from Swedish biopsy charts [14]. We chose to divide patients into four categories according to these requirements:

i) Confirmed IgAN: required mesangial IgA deposits in biopsy record, IgAN as primary biopsy diagnosis, IgAN stated in patient chart, and no data or clinical presentation contradicting IgAN.
ii) Likely IgAN: required mesangial IgA deposits in biopsy record, and *either* IgAN as primary biopsy diagnosis *or* IgAN stated in the patient chart, and no data or clinical presentation contradicting IgAN.
iii) IgAN as Secondary diagnosis: above-mentioned histological requirements for IgAN are met, but IgAN is not likely to be responsible for the main clinical presentation or reason of decline in kidney function.
iv) Not IgAN: when neither clinical nor histological requirements are met.

All patient charts and biopsy reports were reviewed according to a standardized template. We assessed clinical presentation, comorbidities, medications, and laboratory parameters available from the year of the biopsy and one year after the time of biopsy, to differentiate from other plausible diagnoses.

We classified immunofluorescence staining for IgA, C3 and C1q positive if the biopsy chart stated it as weak, moderate, or strong, but as negative if reported as just traces.

### Statistics

We calculated a positive predictive value (PPV) with 95% confidence interval for IgAN diagnosis, merging categories i-ii, but we also provide data when including IgAN as secondary diagnosis (i.e., categories i-iii). Calculations were made using IBM SPSS Statistic software (version 28.0.1.1(15)).

### Ethics approval and consent to participate

The study was approved by the Swedish Ethical Review Authority (Etikprövningsmyndigheten), on the 31st of January 2022, approval number 2021-066629-01. Because this is a strictly register-based study, the requirement of informed consent is not required [15].

## Results

In total 142 IgAN registrations/patients from 29 different nephrology departments in Sweden were randomly selected for the validation. After contacting all primary caregivers, medical charts were received from 140 (98.6%) patients from 27 departments. One patient had the same biopsy registered twice in the SRR, and both versions had been randomly selected, hence the final number of patients undergoing review were 139.

### Clinical characters in IgAN patients

The median age at the time of the biopsy in this subset of 139 patients with a histological diagnosis of IgAN was 46 years (range: 18–85). The male:female ratio was 2.1:1. The median creatinine level at the time of the biopsy was 123 µmol/L and the median eGFR level 51 mL/min/1.73m^2^, with *n* = 80 (57.6%) of the patients having a kidney function impairment corresponding to CKD stage ≥ 3. Hypertension was seen in *n* = 97 (69.8%) of the patients. Proteinuria in *n* = 138 (99.3%) and hematuria in *n* = 132 (94.7%). In 12.9% (*n* = 18) there was a history of purpura consistent with IgAV. No patient had a documented family history of IgAN/IgAV. (Table 1)

**Table 1:** Clinical presentation

Clinical features	*n* = 139
Age (years, median [range]) Male (years, mean) Female (years, mean)	46 [18–85] 46.5 43.3
Sex (male:female n; %)	94:45 (67.6:32.4)
Heredity of kidney disease (n, %)	8/127 (6.3) None for known IgAN
CKD (Chronic kidney disease) stage ≥ 3 (n; %)	80 (57.6)
Hypertension (Blood pressure, BP > 140 and/or > 80 mmHg or Antihypertensive medications) (n; %)	97 (69.8)
Malignant hypertension (BP > x/120 mmHg) (n; %)	5 (3.6)
Proteinuria (U-albumin/creatinine ratio, U-ACR > 3 g/mole) (n; %)	138 (99.3)
Hematuria (Positive dipstick or sediment) (n; %)	132 (94.7)
Nephrotic syndrome (n; %)	8 (5.8)
Purpura (n; %) **Cases classified as IgA Vasculitis** Age, years, median [range] Creatinine (µmol/L; median [range]) eGFR (estimated Glomerular Filtration Rate, mL/min/1.73m^2^; median [range])	19 (13.6) 18 (12.9) 50 [18–85] 86 [55–173] 77 [34–125]
**Laboratory features**	
Creatinine (µmol/L; median [range])	123 [51-1331]
eGFR (mL/min/1.73m^2^; median [range])	51 [5-125]
CKD 1–2 (eGFR ≥ 60) (n; %)	59 (42.4)
CKD 3a (eGFR ≥ 45 and < 60) (n; %)	20 (14.4)
CKD 3b (eGFR ≥ 30 and < 45) (n; %)	33 (23.7)
CKD 4 (eGFR < 30) (n; %)	22 (15.8)
CKD 5 (eGFR < 15) (n; %)	5 (3.6)
Systolic BP (mmHg, mean ± SD)	133.3 (± 15.6)
Diastolic BP (mmHg, mean ± SD)	79.8 (± 9.9)
U-albumin/creatinine ratio (mean ± SD) n; % U-ACR < 30 g/mole, n; % U-ACR > 30 g/mole	135.2 (± 129.4) 28 (20.1) 111 (79.9)
Antineutrophilic cytoplasmic antibodies (n; %)	1/114 (0.9) *
Antinuclear antibodies/anti-dsDNA (n; %)	8/98 (8.2) All anti-dsDNA negative
Serum/Urine electrophoresis with monoclonality (n; %)	1/114 (0.9) **
HIV (n; %)	0/90 (0.0)
Viral Hepatitis (n; %) [B/C]	2/94 (2.1) [1/1]
**Complications post-biopsy**	13/139 (9.4)
Symptomatic hematoma (pain)	10
Macroscopic hematuria	3
Severe complications	0

*Case classified as Not IgAN, **IgG 0.5gr/L, No Bence-Jones proteinuria

### Validation

#### Categories i and ii

Out of the 139 chart validated patients, 107 (77.0%) were categorized as *Confirmed IgAN* (category i) with an unambiguous diagnosis code in the biopsy chart and clear statement of IgAN in the medical records. Another 25 (18.0%) were classified as *Likely IgAN* (category ii). The main reason for being categorized as “Likely” and not “Confirmed”, was scarce material in the biopsy specimen and/or failed immunofluorescence staining. All these patients had a clear statement of diagnosis and clinical presentations corresponding with IgAN described in their medical charts, and in four of the 25 patients, the registration represented the patients’ second (or later) biopsy, further strengthening the IgAN diagnosis. In two patients with Likely IgAN (males, 54 and 42 years), the pathologist suggested that the histological finding was consistent with Secondary IgAN. This is a debated entity and commonly considered as having no significant differences in terms of the histological picture [8]. Nevertheless, both patients had a history of gastrointestinal symptoms and/or surgery (diarrhea from bile salt malabsorption and previous gastric bypass surgery respectively).

#### Categories iii and iv

In four (2.9%) patients, another clinical condition than IgAN was more likely to explain the patients´ symptoms or deteriorating kidney function, even though the histological requirements for IgAN were met. We categorized these as *Secondary diagnosis* (category iii). More specifically, in two patients (males, aged 54 and 64) the exact cause of the kidney failure was unclear, and even though the criteria for IgAN were met there was no proportional affection of the glomeruli in the biopsy specimen compared to the level of creatinine. The third patient, a man aged 72 with histological changes corresponding to IgAN, but additionally had changes associated with his diabetes mellitus diagnosis which also was his primary kidney diagnosis in the medical chart. Finally, the fourth patient, a woman aged 67 with granulomatosis polyangiitis (GPA) in remission, had undergone a biopsy due to a rise in her antineutrophilic cytoplasmic antibody (ANCA) level (1700 E/mL).

**Fig. 1:**
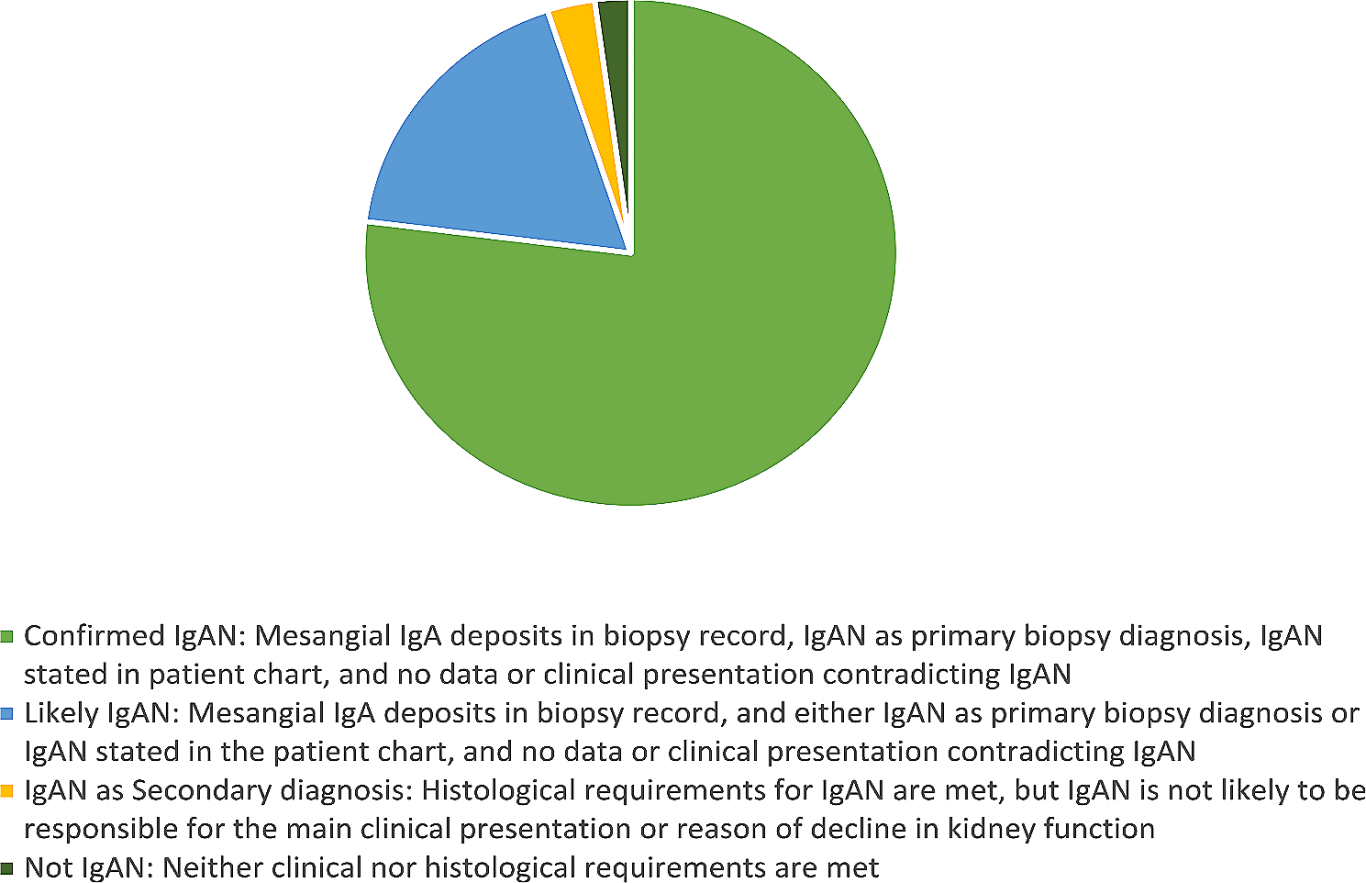
IgAN; IgA Nephropathy

In three out of 139 (2.2%) patients IgAN were not considered to be the correct diagnosis, hence classified as *Not IgAN* (category iv): (1) man aged 81 with pulmonary fibrosis that developed proteinuria after treatment with a VEGF-inhibitor, which is a side effect previously described in case reports [16, 17], (2) woman aged 63 with a complex subset of clinical systematic symptoms not typical for IgAN (including fever, pericarditis, rashes, weight loss), inconclusive biopsy findings and where the treating physician later refuted the IgAN diagnosis, (3) woman aged 42 with relapsing nephrosis, that received treatment and clinically more resembled Minimal Change Disease or Focal Segmental Glomerulosclerosis [18]. (Table 2; Fig. 1)

**Table 2:** Results after medical chart review

	All patients	Confirmed IgAN	Likely IgAN	IgAN as Secondary diagnosis	Not IgAN	Missing or Duplicate data
n (%)	142	107 (75.4)	25 (17.6)	4 (2.8)	3 (2.1)	3 (2.1)
Age (median, years)	46	44	51	65.5	63	42
Male: n (%)	97 (68.3)	72 (67.3)	18 (72.0)	3 (75.0)	1 (33.3)	3 (100.0)
Calender year						
2015	23	15	6	1	0	1
2016	26	20	4	1	1	0
2017	26	24	1	0	0	1
2018	33	26	5	1	1	0
2019	34	22	9	1	1	1

IgAN; IgA Nephropathy

### Additional clinical features

Earlier or ongoing IgA vasculitis (purpura, joint pain and/or abdominal pain), was seen in 18 of 139 (12.9%) patients (Table 2).

In three patients (males, 26, 32 and 49 years) the histological specimen showed IgAN with thrombotic microangiopathy (TMA). All of them had presented with malignant hypertension and/or severe headache. Dialysis was initiated the same year as the biopsy was performed in two of the cases.

In 16 patients, the validation biopsy did not represent the first biopsy. The main reason for having a second biopsy or more was progressively impaired kidney function and/or increased proteinuria in an already diagnosed IgAN, but also scarce material in first biopsy (1 case, category ii), highly elevated ANCA-levels in the patient with GPA in remission (1 case, category iii) and relapsing nephrosis (1 case, category iv).

### Biopsy findings

The biopsy charts contained reports on mesangial IgA deposits in 132/132 (100%) patients with IgAN, mesangial hypercellularity or proliferation was mentioned in 102/132 (77.2%), and immunofluorescence staining for C3 was positive in 98/132 (74.2%).

In 27 (19.4%) out of the 139 biopsies the pathologist had noted a MEST score. Out of these 27 cases, 26 (96%) were classified as *Confirmed* or *Likely IgAN (category i and ii*)., even in the two cases where the score was M0 E0 S0 T0. In the last case, with the score M1 E0 S0 T0, the IgAN diagnosis was refuted by the treating clinician (*Not IgAN*, *category iv*).

C1q positivity was reported in 20.5% (27/132) of all the patients, and in 2/18 (11.1%) of the patients classified as IgAV (Table 3).

**Table 3:** Histology and biopsy findings

Histology	n (%)
Mesangial IgA-deposits	132/132 (100)
Mesangial hypercellularity/proliferation	102/132 (77.2)
Mesangial C3 deposits	98/132 (74.2)
Mesangial C1q deposits	27/132 (20.5)
Glomerulus in specimen (mean, [range])	20 [2–75]

### Positive predictive value (PPV) for IgAN diagnosis in the SRR

Our chart validation found that 132 of 139 patients belonged to category *Confirmed* or *Likely IgAN (i or ii)*, i.e. had a clinical and histopathological diagnosis of IgAN/IgAV, yielding a PPV for a correct diagnosis of 95% (95% CI 90–98%). When we also included IgAN/IgAV as a *Secondary diagnosis (category iii)* the PPV increased to 98% (95% CI 93–99%).

### Complications

Complications from the kidney biopsy were reported in 13/139 patients. The most common complication was flank pain that led to prolonged in-patient care and/or need for extra radiological examination. Macroscopic hematuria was identified in 3 patients but there were no reports of severe complications.

### Completeness

**Fig. 2:**
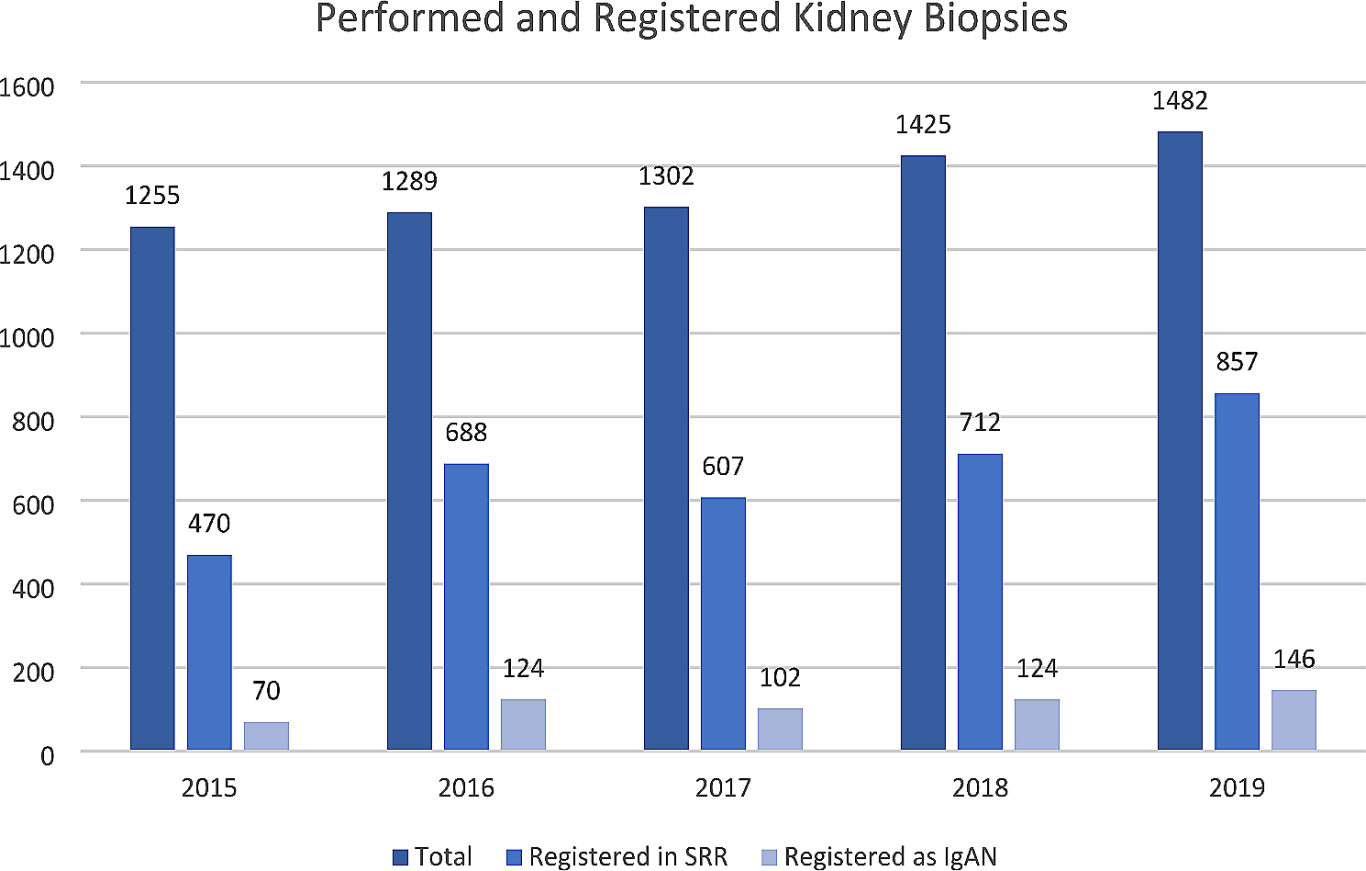
IgAN; IgA Nephropathy, SRR; Swedish Renal Registry

Registering biopsy data in the SRR was introduced in 2015 and they have increased their completeness from 37 to 58% over the first 5 years, based on numbers from the pathology departments on annually evaluated kidney biopsies compared to the total number of biopsy registrations. There has been a slight increase in the total number of kidney biopsies sent for histopathological examination in Sweden during the corresponding years, from 1255 in 2015 to 1482 in 2019, but the percentage of IgAN/IgAV diagnoses has remained stable between 15 and 18% during the whole period (Fig. 2).

## Discussion

Sweden and the Nordic countries are internationally well-known for their unique population-based registries, both mandatory nationwide health care registries and national quality registries, such as the SRR. Continuous validation of these registries is an important feature necessary to ensure reliable data, and hence indispensable for both improvement and monitoring of clinical quality as well as to lay the foundation of good epidemiological data for research purposes. Additionally, it is mandatory for all tax-funded Swedish qualitative registers to report internal quality validation on a regular basis to continue receive funding. Validating IgAN is also important since it opens up for more studies on etiology and prognosis in patients with biopsy verified IgAN. Earlier research suggest that these patients are at increased risk of death [19], inflammatory bowel disease [20], ischemic heart disease [21], but also adverse pregnancy outcome [22].

### Comparison to previous literature

The clinical presentation of IgAN is similar to that in other types of glomerulonephritis: microscopic hematuria and proteinuria in varying degrees, but the disease also has some unique histological features with characteristic immunocomplex deposits and positive immunofluorescence staining.

We found a high PPV for a histological IgAN diagnosis with a corresponding clinical IgAN diagnosis in patients registered in the SRR after having performed a kidney biopsy. The PPV was further increased from 95 to 98% when we also included cases with IgAN as secondary kidney diagnosis. Our findings are very similar to those earlier reported by Jarrick et al., who found a PPV of 95% (95%CI 92–99%), with IgA deposits in 84% of the cases, mesangial hypercellularity in 76% and C3 deposits in 89% [14].

Previous studies have indicated that the classical complement pathway protein, C1q, could serve as a marker for more severe IgAN and correspondingly worse prognosis [23–25]. Jarrick et al. included description of C1q depositions in their subset of IgAN patients and reported a frequency of 12%, in comparison we found C1q positivity in 20.5% of cases. The median age was similar between C1q positive cases and the total cohort (45 years vs. 46 years respectively) and so was median creatinine (133.5 µmol/L vs. 123 µmol/L) at the time of the biopsy.

The underlying reasons for the lower frequency of C3 deposits and higher C1q deposits in our cohort are unknown.

### Previous validation

Jarrick et al. [14] validated Swedish biopsy chart results with clinical presentation, whereas we validated actual registered data in the national quality register SRR, thus data that will be used in future research projects. Compared to Jarrick et al. [14], our cohort of validated patients also have a higher frequency of hypertension (69.8% vs. 56%), higher mean creatinine (123 µmol/L vs. 104 µmol/L) and a lower mean eGFR (51 mL/min/1.73m^2^ vs. 75 mL/min/1.73m^2^). In contrast to Jarrick et al., our cohort did not include any children.

### Strengths and limitations

Our validation study has several important strengths such as: (i) the high percentage (98%) of medical charts obtained for review, (ii) that charts were selected from a large number of Swedish nephrology clinics (*n* = 29) likely decreasing selection bias and (iii) the detailed information including laboratory data, original biopsy record and medical charts, that enabled us to assess the full clinical picture of the included patients.

A limitation in the study is that a MEST-C score was only reported in 19.4% of the biopsy statements. Information on endocapillary hypercellularity, segmental glomerulosclerosis, tubular/interstitial atrophy and the presence of crescents was generally included in the descriptive texts, but not graded according to the scoring system. However, since the score is foremost a prognostic marker [26, 27], we argue that the lack of a score was not crucial for the IgAN diagnosis as such. Even so, we would like to encourage the pathologists to use the MEST-C score more frequently and systematically, and the SRR to consider incorporating this variable in the registry, so it can be used in future research on progress and outcome predictions.

Another limitation is that we had to limit the follow up to one year before and after diagnosis. Further, it is possible that pivotal information known to the physician may not have been reported in the medical charts available for review.

## Conclusion

Out medical chart validation established a high PPV (95%) for clinical IgAN in patients with an IgAN biopsy registered in the SRR. Clinical characteristics of the evaluated patients were consistent with previous reports of IgAN patients. The validated biopsy data in the SRR will be a valuable resource in future IgAN research.

## Data Availability

The datasets used and/or analyzed during the current study available from the corresponding author on reasonable request.

## Abbreviations

ANCA: Antineutrophilic cytoplasmic antibody
BP: Blood pressure
CI: Confidence interval
CKD: Chronic kidney disease
eGFR: Estimated glomerular filtration rate
GPA: Granulomatosis with polyangiitis
IgAN: IgA nephropathy
IgAV: IgA vasculitis
OR: Odds ratio
PPV: Positive predictive value
SD: Standard deviation
SNOMED: Systematized Nomenclature of Medicine
SLE: Systemic lupus erythematosus
SRR: Swedish Renal Registry
TMA: Thrombotic microangiopathy
